# Global Aid Cuts and Local Health Consequences in Nakivale Refugee Settlement, Uganda

**DOI:** 10.1001/jamanetworkopen.2026.15000

**Published:** 2026-05-26

**Authors:** HaEun Lee, Camilla Bjelland, Kay Wallace, Donath Asiimire, Fred Sheldon Mwesigwa

**Affiliations:** 1 School of Nursing, University of Michigan, Ann Arbor; 2 Medical School, University of Michigan, Ann Arbor; 3 Department of Economics, Statistics, and Tourism Management, Bishop Stuart University, Mbarara, Uganda; 4 Department of Humanities, Bishop Stuart University, Mbarara, Uganda

## Abstract

**Importance:**

The abrupt withdrawal of humanitarian aid in early 2025 has destabilized health systems across sub-Saharan Africa, yet little is known about the frontline realities of these cuts in refugee-hosting settings.

**Objective:**

To explore health care practitioners’ perspectives and experiences of how reductions in global health funding have affected services and refugee health and to identify practitioner recommendations for sustaining care.

**Design, Setting, and Participants:**

This qualitative study used semistructured, in-depth interviews conducted in July 2025 at 6 health facilities within Nakivale Refugee Settlement, Uganda. Participants were health care practitioners representing diverse cadres and facility sizes.

**Exposure:**

Reduction of humanitarian aid.

**Main Outcomes and Measures:**

The primary outcomes were health care practitioners’ perspectives on the impacts of funding reductions, including changes in service delivery, supply availability, staff conditions, and anticipated future health trends, as well as their recommendations to mitigate harms. Rapid qualitative techniques and thematic analysis were used to analyze responses to obtain actionable implications systematically and efficiently.

**Results:**

The 26 participants (mean [SD] age, 30.92 [3.52] years; 16 men [61.5%]) had a mean (SD) duration in practice of 6.80 (2.65) years, with a mean (SD) of 3.09 (1.92) years in Nakivale. Interviews yielded 4 overarching themes and 18 subthemes: (1) reductions in health care services (HIV and tuberculosis services, nutritional support, maternal health, immunization adherence, inpatient and outpatient care, and perceptions of service availability), (2) supply shortages (medical supplies, transportation fuel, and household support), (3) deteriorating staff conditions (staff reduction, workload and burnout, and resilience), and (4) future predictions and recommendations (disease patterns, migration patterns, bridging the funding gap, and general settlement conditions).

**Conclusions and Relevance:**

This qualitative study examined the cascading outcomes of humanitarian aid withdrawal on refugee health care through the voices of frontline practitioners. Their testimonies underscored urgent priorities for restoring and retargeting aid—investing in high-impact levers, strengthening local leadership, and advancing policy reforms—to safeguard refugee health and system resilience, while reminding us of the shared human stakes of humanitarian policy and the global responsibility to act.

## Key Points

**Question:**

What are the perspectives and experiences of health care practitioners in Nakivale Refugee Settlement in Uganda following substantial reductions in humanitarian funding?

**Findings:**

In this qualitative study of 26 health care practitioners—including physicians, midwives, nurses, and laboratory staff—participants described profound disruptions to health care services, supply chains, and staffing conditions, and discussed their predictions and recommendations for sustaining care.

**Meaning:**

Restoring and strategically targeting humanitarian aid is essential, with investments prioritized for high-impact leverage points, including prenatal nutrition and immunization incentives, fuel for transport, supply redistribution, and salaries for health care practitioners, while building long-term resilience through refugee-led organizations and policies that integrate refugees into civilian labor markets.

## Teaser

This qualitative study examines health care practitioners’ perspectives and experiences of how reductions in global health funding have affected services and refugee health and identifies practitioners’ recommendations for sustaining care.

## Introduction

The global landscape of humanitarian and development assistance is undergoing a profound transformation due to drastic funding reduction by multiple donors.^1,2^ Historically, Development Assistance for Health peaked at $80.3 billion in 2021 and steadily decreased to $49.6 billion in 2024.^3^ In 2025, the aid was expected to decline to $38.4 billion, an amount last seen in 2009.^3^ The US accounted for 43% of all government funding donated by countries to humanitarian systems in 2023, with US Agency for International Development (USAID) managing more than $35 billion in 2024.^4^ However, an executive order signed on January 24, 2025, dismantled USAID programs, canceling 90% of contracts and grants by February and reducing programs by 83%.^1^ Other donors, including the United Kingdom, Germany, and Canada, have also enacted major cuts, collectively decreasing Development Assistance for Health aid by over €2 billion.^5,6^ The projected consequences are dire: 14 million additional deaths by 2030, including 4.5 million children younger than 5 years, alongside 11.7 million women losing access to reproductive health care, resulting in 4.2 million unintended pregnancies and over 8000 maternal deaths in 2025 alone.^7^

Uganda, which hosts close to 2 million refugees—the largest population in Africa—is facing an acute crisis as a result.^8^ USAID cuts represent a $307 million loss, equating to a 66% reduction in programs.^9^ In addition, the country has been strained by influx of refugees with a 173% increase in refugee population since early 2025.^10^ Uganda’s Ministry of Health faces a $114 million budget gap, risking closure of all dedicated HIV and AIDS and tuberculosis (TB) clinics, with supply chain disruptions for antiretroviral therapy and malaria test kits already reported.^11,12^

In Nakivale refugee settlement—the oldest refugee settlement in Africa and home to more than 274 000 refugees—the consequences are severe.^13^ Although existing literature documents global funding shifts^1,3,14^ and mortality projections,^3,12,14,15,16,17,18,19,20,21^ there is little in-depth qualitative research from health care practitioners themselves describing their experiences living and working through the unprecedented funding conditions of the mid-2020s. This study explores practitioners’ lived experiences, challenges, and adaptive strategies on the frontlines of Nakivale, providing a ground-level perspective that complements broader impact assessments.

## Methods

This qualitative study was approved by the institutional review boards of the University of Michigan, Bishop Stuart University, and Uganda National Council for Science and Technology. Additional approval was obtained from Uganda’s Ministry of Health to conduct research in the refugee settlement. Written informed consent was obtained from all participants. The study adhered to Consolidated Criteria for Reporting Qualitative Research (COREQ) reporting guidelines.^22^

We recruited health care practitioners in Nakivale refugee settlement using maximal variability sampling, defined by recruitment of various practitioner roles across the 6 health facilities and within all 3 administrative zones of Nakivale. Through the 4 health care practitioners with whom we had relationships from prior research projects, we recruited additional participants via snowball sampling. This ensured that the findings reflect various levels of clinical responsibility and the unique resource constraints of different sectors within the settlement. This study was guided by phenomenological theory^23^ to prioritize the professional experiences of practitioners and a socioecological lens^24^ to map how macro-level funding cuts translated into systemic operational failures. We applied rapid qualitative techniques with deductive and inductive thematic analysis to capture anticipated and emergent themes.^25,26^

### Characteristics of the Study Team

The team comprised 5 authors: 4 women (2 White US individuals, 1 South Korean individual, and 1 Ugandan individual) and 1 Ugandan man. Three had extensive experience as researchers with refugee populations (H.L., D.A., and F.S.M.) including Nakivale, and 2 were new (C.B. and K.W.) to the context as trainees.

### Setting

Nakivale is the oldest refugee settlement in Africa and the largest in Uganda, with more than 274 000 individuals as of March 2026.^27^ It predominantly houses individuals from Congo (77.8%), Burundi (12.9%), and Rwanda (5.7%).^27^ Women and children represent 74.0% of the population, with an approximately equal distribution of male and female individuals overall.^27^ The settlement is divided into 3 zones—Base Camp, Rubondo, and Juru.^27^ Base Camp houses 71 251 individuals, Juru 100 008, and Rubondo 106 261.^27^ We interviewed practitioners from 6 health care facilities: Rubondo and Ruhoko in Rubondo zone, Juru and Kibengo in Juru zone, and Kabanza and Nakivale health center in Base Camp zone.

### Recruitment and Sampling

Eligible participants were adult health care practitioners (aged ≥18 years) with at least 1 year of experience in Nakivale health facilities and proficiency in English. Although English and Swahili are both official languages of Uganda, English has a longer history of official status and remains the primary language of formal education. Recruitment flyers were distributed via WhatsApp and SMS through leads at 6 facilities to ensure diversity in role, experience, and gender. We aimed to recruit 4 to 6 practitioners per facility (up to 30 total) or until data saturation (see interview procedures). No participant whom we recruited or was referred refused to participate or dropped out during the interview.

### Interview Procedures

Two researchers (H.L. and C.B.) both trained in qualitative methods conducted one-on-one interviews in private spaces at health facilities. Some of the spaces were enclosed rooms and some were open spaces with other nonparticipants present, but not close enough to hear the conversation. Description of the project was provided, and consent and demographic data were recorded in REDCap, with manual entry as needed per limited data coverage. Semistructured interview guides (eAppendix 1 in Supplement 1) were used to conduct the interviews (15-30 minutes). Interviewers wrote field notes during each interview, and all interviews were audio-recorded. To ensure data saturation, the interviewers conducted iterative debriefing sessions after each health facility visit. These sessions involved a thematic review of field notes to determine whether new perspectives were still emerging. Saturation was confirmed when a stopping criterion of 3 consecutive interviews yielded no new thematic categories,^28^ indicating that the breadth of practitioner experiences had been captured. Each participant received 50 000 UGX (approximately $14 US) as a token of appreciation of their time (professional salaries of health care practitioners range from 960 000 to 3.5 million UGX per month). All interviews were conducted between July 7 and 9, 2025.

### Statistical Analysis

Descriptive analysis was run using StataSE version 18 (StataCorp). We used rapid qualitative techniques and thematic analysis.^25,26^ Data were entered directly into structured summary templates aligned with the interview guide.^26^ Deductive and inductive approaches were combined: a deductive framework structured initial coding, and inductive coding allowed for unanticipated issues to emerge.^26^

Two researchers (H.L. and C.B.) summarized each other’s interviews into structured templates; a third (K.W.) drafted the codebook and applied the drafted codebook, compiling data into an Excel (Microsoft) matrix. Then, D.A. and F.S.M., who independently reviewed audio recordings, confirmed and added additional codes. All authors jointly confirmed the final themes and subthemes. We did not calculate interrater reliability. Instead, we prioritized consensus-based approach through frequent team meetings and cross-checking until agreement was reached between all 5 authors.^25,26^ Codes present in fewer than 5 interviews were merged or dropped (eAppendix 2 in Supplement 1). The drafted results were shared with 3 participants, each from different health facilities, for participant checking.

## Results

Table 1 presents demographic characteristics of the 26 participants (mean [SD] age, 30.92 [3.52] years; 16 men [61.5%]). Participants had a mean (SD) of 6.80 (2.65) years of professional experience and 3.09 (1.92) years of practice in Nakivale. The sample included 11 physicians (42.3%), 6 midwives (23.1%), 6 nurses (23.1%), 2 laboratory technicians (7.7%), and 1 health assistant (3.8%). Practitioners were drawn from 6 facilities: Kibengo, Juru, Nakivale, Rubondo, Rukoho, and Kabanza. Four major themes and 18 subthemes were identified. Exemplar quotes are presented in Table 2.

**Table 1.** Demographic Characteristics of Study Participants

Characteristics	Participants, No. (%) (N = 26)
Age, mean (SD), y	30.92 (3.52)
Gender	
Women	10 (38.5)
Men	16 (61.5)
Facility	
Kibengo	2 (7.7)
Juru	3 (11.5)
Nakivale	7 (26.9)
Rubondo	6 (23.1)
Ruhoko	4 (15.4)
Kabazana	4 (15.4)
Health care practitioner type	
Physician	11 (42.3)
Midwife	6 (23.1)
Nurses	6 (23.1)
Laboratory technicians and assistants	2 (7.7)
Health assistants	1 (3.8)
Time as health care practitioner, mean (SD), y	6.80 (2.65)
Time in Nakivale refugee settlement, mean (SD), y	3.09 (1.92)

**Table 2.** Themes and Subthemes of 26 Interviews With Quantification of Frequency

Themes, subthemes, and participant location^a^	Quotation
Reductions in health care services	
HIV and tuberculosis (n = 17 interviews)	
Nakivale	“Deduction in staffing in the HIV and TB department. And that has caused us to not be able to monitor the prognosis about these diseases.”
Nutrition (n = 19 interviews)	
Kibengo	“When [WFP] stopped, we experienced an exponential increase within the malnutrition cases.”
Juru	“We had community nutrition services…all of them were closed. But one of the services at the facility level has come back. But it is targeting those [children] who are severely malnourished, not moderately malnourished. When you get those that are moderately malnourished, they are paused a bit. Once they turn into severe [malnourishment], then they are attended [to].”
Maternal care (n = 12 interviews)	
Ruhoko	“Mothers used to receive Soya in January, since the WFP stopped funding this, so mothers failed to turn up.”
Kibengo	“It encouraged the mothers who were pregnant to come for ANC as early as possible. When a woman missed her period, she came immediately because she knew that she was getting that CSB, the porridge.”
Kabazana	“People come for antenatal services for the first time at 7 months or 8 months.”
Children’s immunizations (n = 5 interviews)	
Kibengo	“They first look at the vaccination card of the baby and then they give you the service, the porridge. It encouraged them to come for services, the immunization.”
Inpatient and outpatient services (n = 12 interviews)	
Rubondo	“With the reduced funding, we cannot serve our communities the way we were doing it at first. In our family planning, we cannot do the community outreaches we were doing before.”
Juru	“There are some facilities where those services [inpatient admissions] were laid off because of human resources. You cannot operate outpatient department plus maternity department plus admissions. So admissions has to close in one of those facilities.”
Juru	“It is easier for [new arrivals needing inpatient care] to feed themselves when they are in Juru than in Nakivale.”
Contraceptive (n = 8 interviews)	
Rubondo	“(Previously people thought) They are children of UN, I am free to produce because they were getting money per head one could say, ‘The more children I have, the more money I get.’ But now the money is not there, to feed them, to provide for them. They were getting 30,000 UGX (8.4 USD), but it kept on reducing. I think many of them get no money at all now.”
Juru	“There are some hardships, so family planning uptake has been high.”
Perception of availability (n = 5 interviews)	
Ruhoko	“(People think) Since the US is no longer giving us aid, the medicines are not there. Some have stopped coming, others have gone months without medication.”
Kibengo	“When our mothers heard about that Trump thing, they got scared and started disappearing. We kept telling them, the drugs are there, it is only the staff that went. We tell them, ‘There are other people to support you, please come back to the facilities.’”
Supply shortages	
Medical supplies (n = 16 interviews)	
Nakivale	“Essential medicines are out of stock. Some meds are supplied in few quantities; others are not there at all. Antihypertensives: enalapril, captopril, lisinopril, all of them are out of stock. Children’s meds are out. What supports us is the drugs from the government.”
Juru	“We are receiving less drugs, about 40-50% of the drugs originally received. You tell your patients that we don’t have them, you have to go buy it yourself.”
Transportation fuel (n = 6 interviews)	
Nakivale	“Most of our vehicles are parked. Fuel also was reduced.”
Nakivale	“Every vehicle is allocated a certain quantity of fuel, so when the month is coming to an end, you find that some cases such as emergencies, they have blown off the fuel, they put a hold on the referrals. They say, ‘make only necessary referrals.’ You go backwards, you can see the issues are really coming from the funding cuts.”
Household support (n = 14 interviews)	
Rubondo	“People that used to receive money are no longer receiving money.”
Rubondo	“In the villages, [I] hear some say, ‘We are going to die, so now that they have cut the food.’”
Ruhoko	“People are almost starving because their rations were reduced. People can’t sustain their families; there is not enough food.”
Deteriorating staff conditions	
Staff reduction and rehire (n = 26 interviews)	
Rubondo	“Almost 50% laid off, cut across all staff, both technical staff and support staff. Doctors, midwives, clinical officers, nurses. Some are home.”
Nakivale	“You have been having a job and you are told to go home. Anticipate people to have the mental issues, comes with depression, depression comes with suicidal tendencies.”
Workload and burnout (n = 24 interviews)	
Kibengo	“Workload was too much. The staff was very few. With service delivery, when staff if very few, you just work to finish you cannot give ample time for each patient. There are very many and they need your services, and you are too stressed out and you want to finish up and yet you need to work on all of them because we do not chase away patients. Of course, if there are 100 and you are 2 midwives or you are 1. You cannot go on leave, you can’t just leave one midwife.”
Nakivale	“Expect the staff to burnout; when they burn out, they may give the wrong medication; maybe he falls sick because of fatigue, and definitely the service delivery will be impacted.”
Helplessness (n = 6 interviews)	
Kibengo	“You find the mother has nothing to dress up when she is done with labor, with delivery, the babies’ clothes are not there. These are mothers that come without anything to eat, even while they are laboring.”
Nakivale	“We maintain, we give them what we have, knowing very well it will not do anything. Someone is convulsing and you give the anticonvulsant, and it isn’t working, and you wish for a better alternative, but it is not there. You cannot tell the patient to go and buy. They cannot afford.”
Resilience and commitment (n = 13 interviews)	
Nakivale	“The new people who enter our country, most of these people are already in a bad condition…. Their psychological, mental state is compromised…. So they need at least tender, loving care. And for us to offer this tender, loving care, we need adequate human resources.”
Nakivale	“We are hopeful, we have to work in the means that is available, ensuring that we serve our clients with the minimum package that we have provided to us.”
Ruhoko	“[I started working here because] I had love for the patients…. People were suffering and I had to do something…. I have persisted because I am passionate about what I do.”
Future predictions and recommendations	
Disease patterns (n = 12 interviews)	
Rubondo	“Malaria cases are increasing, these people come, and they don’t have malaria prevention strategies like mosquito nets.”
Migration patterns (n = 12 interviews)	
Juru	“By the end of this month we will have some people going home [back to home country] again.”
Kibengo	“If someone is running off from the war, and they reach this side, and they are like, ‘Oh, it is like this; I would rather die.’”
Nakivale	“As they go back, they may even die along the way.”
Needs and recommendations (n = 20 interviews)	
Ruhoko	“I am hopeful that things will be better, that funding will come. Of course, we are prayerful. Our government and stakeholders have learned something, and they will prepare themselves more to make sure that the healthcare system does not struggle in case of lack of funding.”
Settlement conditions (n = 14 interviews)	
Kabazana	“Need more community awareness, need to allow the younger generation to do more training so they remain a self-supported community, not waiting for the funding.”
Juru	“What is going to happen, in the coming year if UNHCR withdraws completely, it is going to be bad. We shall have many outbreaks…. [And] remember the women will be producing and not getting enough to eat. So, fights between the woman and their husband, GBV cases, we expect them to increase. And death will be coming. Conflict in the communities. People will kill themselves.”
Ruhoko	“If things don’t improve, the refugee setting will suffer…. They are not able to buy medicine, or food, or sanitation. So I think their lives will be at risk.”

Abbreviations: ANC, antenatal care; CSB, corn soy blend; GBV, gender-based violence; TB, tuberculosis; UN, United Nations; UNHCR, United Nations High Commissioner for Refugees; WFP, World Food Programme.

^a^ Numbers of interviews indicate the number of interviews the subtheme has been mentioned.

### Theme 1: Reductions in Health Care Services

Health care workers described reductions in health care services related to HIV and TB, nutrition, maternal health, children’s immunizations, inpatient and outpatient, and contraception. HIV and TB services were among the most severely affected. Entire departments were closed, halting testing, treatment, contact tracing, and outreach. The deduction in staffing, “caused us to not be able to monitor the prognosis about these diseases” (Nakviale). Although some services were partially absorbed by outpatient and maternity departments, this redistribution was inconsistent and burdensome. Practitioners voiced deep concern about worsening outcomes among existing patients and increasing HIV and TB prevalence without dedicated staff and services.

The reduction of World Food Programme’s nutrition support had a pervasive impact. Previously, corn soy blend, also known as *soya*, was distributed to malnourished children, children presenting for routine immunizations, and pregnant and lactating women. These programs were largely suspended or restarted at minimal capacity. Practitioners observed, “an exponential increase within malnutrition cases” (Juru), particularly among children younger than 5 years who progressed from moderate to severe malnutrition after the suspension of soya supplements previously provided to moderately malnourished children. They also noted increases in maternal malnourishment, anemia, and stillbirths, attributing these to declining nutritional support.

Maternal health, including antenatal, delivery, and postnatal services, were destabilized. Without nutrition incentives provided during these visits, women delayed antenatal visits until later trimesters and decreased facility-based deliveries and postnatal visits. Staffing shortages and supply gaps—such as the mama kits and mosquito nets once used to also encourage facility-based care—were linked to increased home deliveries and decreased adherence to recommended antenatal care contacts.

Immunization adherence also fell after incentives were withdrawn. Practitioners reported outbreaks of vaccine-preventable diseases, such as measles, and expressed concern about diminished capacity to track and manage outbreaks proactively. Cuts also reshaped inpatient and outpatient care. Staff reductions closed entire wards, eliminated triage systems, and forced patients to travel long distances for hospitalization. Outpatient services, including health education and village screening, were curtailed.

Participants gave mixed perspectives on contraception. Some reported adequate supplies but persistently low uptake, whereas others believed women were utilizing family planning more given household resource constraints. Beyond service changes, perceptions of service availability also shaped care-seeking. “People think since the US is no longer giving us aid, the medicines are not there. Some have stopped coming, other have gone months without medication” (Ruhoko), even when drugs remained in stock. Without outreach capacity, practitioners feared these perceptions would further decrease utilization and exacerbate illness.

### Theme 2: Supply Shortages

Supply shortages were also heavily impacted. Health care workers described changes in medical supplies, fuel, and household support. Shortages of medical supplies were widespread. Facilities ran short of HIV and TB medications, anticonvulsants, antihypertensives, diabetes drugs, analgesics, and antibiotics: “Essential medicines are out of stock. Some meds are supplied in few quantities” (Nakivale). Shortages also included HIV self-test kits, thermometers, gloves, and personal protective equipment, while broken equipment often went unreplaced. Shortfalls in women’s health supplies, including menstrual products, further restricted service delivery.

Transportation fuel rationing was described as equally damaging. Ambulance allocations were cut by half or more, limiting referrals to only the most critical emergencies and leaving patients stranded late in the month. “Most of our vehicles are parked [and not in use]” (Nakivale). Reductions also crippled redistribution of medications between facilities, a practice previously coordinated by USAID to mitigate local shortages.

Cash stipends and food rations for household support diminished sharply, even among new arrivals, with many households reclassified as ineligible. Among those still receiving aid, monthly stipends dropped below 13 000 UGX (approximately $3.65 US), far from sufficient to cover food or medicines. Practitioners linked these changes to reduced access to essential goods, such as medicines and food, and a broader decline in refugee health and resilience.

### Theme 3: Deteriorating Staff Conditions

Reduction in aid also created deteriorating staff conditions. Participants described staff reduction with minimal rehiring, increased workload and burnout, and helplessness, but simultaneously described resilience and commitment to serving their refugee patients. As one midwife noted, “No matter the time, you might also become a refugee…treat them the way you wish someone would treat you.” Participants universally reported large-scale staff reduction across cadres, from doctors and midwives to laboratory technicians, ambulance drivers, and nutritionists. Rehiring occurred unevenly, often through short-term contracts, training roles, or volunteer placements, but these efforts did not restore original capacity. Staff described heightened anxiety about job insecurity and further cuts. Remaining staff faced rising patient loads, longer hours, and responsibilities outside their training, leading to high workload and burnout. A participant described, “When staff are very few, you just work to finish. You cannot give ample time for each patient” (Kibengo). These conditions also increased wait times. Many described working beyond capacity without additional compensation, contributing to stress and burnout. Practitioners expressed helplessness at being unable to treat patients adequately. They described diagnosing conditions without the medications to treat and noted, “You cannot tell the patient to go and buy. They cannot afford” (Nakivale). This mismatch between professional skills and available resources fostered feelings of helplessness and moral distress.

Despite challenges, many emphasized resilience. Practitioners spoke of compassion for patients arriving in “bad condition. Their psychological and mental state compromised,” and described their determination to provide “tender, loving care” (Nakivale) despite systemic constraints. They viewed their work as essential and refused to turn patients away.

### Theme 4: Future Predictions and Recommendations

Health care workers shared their future predictions in disease patterns, refugee migration patterns, and general conditions in the settlement, and recommendations for bridging the funding gap. Participants predicted worsening disease patterns leading to higher morbidity and mortality if current budget cuts continued, particularly among children younger than 5 years, pregnant women, and people living with communicable and noncommunicable diseases, including but not limited to malaria, HIV, TB, diarrheal diseases, respiratory infections, anemia, and malnutrition. Practitioners also feared that delays in seeking care, combined with practitioner burnout and weakened health care capacity, would further weaken the health of Nakivale refugees.

Participants also emphasized changes in migration patterns. Rising refugee arrivals from Congo creating additional pressures, as new arrivals often presented with extensive needs and required comprehensive health assessments. At the same time, some refugees were returning home: “If someone is running off from the war, and they reach this side, and they are like, ‘Oh, it is like this; I would rather die’” (Kibengo).

Practitioners consistently called for restoration of funding and staffing, noting that cuts seemed to be unidirectional and rarely reversed. Although some emphasized the responsibility of international donors, others urged the Ugandan government to invest more substantially in refugee health and reduce dependency on external aid. Participants recommended closer integration between nongovernmental organization and government services to promote sustainability, although many doubted that financial independence from external support was achievable. Staff restoration and stronger support for health care systems were identified as essential to maintaining care.

Finally, participants emphasized that aid cuts affected not only health but also broader settlement conditions, including gender-based violence, mental health, child protection, and safety. These issues, they noted, were deeply interconnected with health. To mitigate impacts, they shared about the “need [for] more community awareness, outreach about available services, and the use of religious and community leaders to share information” (Kabazana). Longer-term suggestions included refugee skills training and language learning programs to build refugee capacities.

## Discussion

This qualitative study illustrates the far-reaching consequences of the humanitarian funding withdrawal. Practitioners’ testimonies reveal how decisions taken at the global levels reverberate directly into clinical practice, altering service delivery, supply chains, staffing, and future health trajectories. Their perspectives converge with national and international reports on USAID and related freezes,^1,3,29^ but add crucial on-the-ground detail about how vulnerable populations, particularly children younger than 5 years, pregnant and lactating women, and people living with HIV and TB, are disproportionately affected. Participants predicted worsening morbidity and mortality from both communicable and noncommunicable diseases, coupled with rising malnutrition, anemia, and weakened preventive services, reiterating findings from other literature.^3,12,14,15,16,17,18,19,20^ They also highlighted the broader deterioration of settlement conditions, including increased gender-based violence, crime, and mental health distress.^1,16^ Participants called for multisectoral responses that integrate health with psychosocial support, protection services, and community safety.^5,16,30^

Nutrition-linked incentives long underpinned early antenatal booking and child immunization uptake, and their removal immediately disrupted preventive care.^20^ Practitioners described later antenatal initiation, more frequent home deliveries, and decreased adherence to vaccination schedules, all of which increase preventable morbidity and mortality. Although the sustainability of incentive-based approaches has been debated in policy circles,^31^ evidence from Nakivale demonstrates their immediate value in fragile contexts where refugee populations face extreme vulnerability and lack alternative forms of support. Therefore, restoring nutrition and immunization incentives is a vital policy priority, consistent with World Health Organization 2016 guidelines recommending balanced supplementation in undernourished populations to reduce stillbirths and small-for-gestational-age births.^32^

Cuts to medical supplies and transportation fuel also had cascading effects. Shortages of essential medications, from HIV and TB drugs to pain management and chronic disease therapies, were compounded by reductions in fuel allocations for ambulances and redistribution vehicles. These cuts crippled referral systems and halted intradistrict transfers that once buffered local shortages. These accounts reveal the critical role of fuel in sustaining care delivery—a connection rarely emphasized in higher-level summaries but evident on the ground. Without transport, patients cannot be moved, supplies cannot be redistributed, and outreach cannot occur. Policy responses must, therefore, acknowledge fuel as an essential health input and ensure that it is adequately funded and protected from volatility.

Staffing emerged as another critical axis of disruption. Layoffs across cadres, inconsistent rehiring, and reliance on volunteers left remaining practitioners working untenable shifts, often across expanded scopes of practice. These conditions not only undermined service quality but also fostered deep moral distress as practitioners diagnosed conditions they could not treat, advised patients to buy medications that were unaffordable, and watched preventable deterioration unfold. At the policy level, these findings underscore the need for emergency aid packages that explicitly include human resources for health.^1^ Restoring staff, securing contracts, and providing fair salaries are not supplementary but foundational to system recovery.

Several broad sets of policy implications emerge from these findings. The first concerns the restoration and retargeting of humanitarian aid toward health sovereignty. Bridge financing from multilateral actors, such as the Global Fund, Gavi the Vaccine Alliance, and the World Bank, is urgently needed to stabilize essential programs. However, the goal should not be to simply restore funding and allocate strategically to leverage points with the greatest multiplier effects—such as prenatal nutrition, immunization incentives, fuel for transport, and salaries for health care practitioners—but to also accelerate self-reliance. This movement is championed by the Africa Centres for Disease Control and Prevention via The Durban Promise, which urges African nations to manufacture, regulate, and procure their own medical countermeasures while financing their health needs through domestic wealth.^33^ For example, Nigeria’s recent federal approval of $200 million (approximately 340 billion Naira) designed specifically to cushion shortfalls caused by US health aid cuts,^34^ alongside the launch of the Codix Bio facility capable of producing 140 million HIV and malaria test kits annually,^35^ provides examples of potential roadmaps for Uganda’s leadership to work toward self-reliance. This transition is best operationalized through the Humanitarian Development Peace Nexus framework, which provides a roadmap for building resilience in fragile settings by integrating emergency relief with long-term development stability.^36^

At the same time, recognition of the resilience and commitment of frontline practitioners underscores the importance of channeling funds toward local actors, particularly refugee-led organizations, which are already sustaining services in the absence of external support.^3,12,14,15,16,17,18,19,20^ Despite strains, resilience was consistently evident. Practitioners emphasized empathy, persistence, and a refusal to abandon patients. Their testimonies highlight the importance of investing in refugee-led organizations, which often sustain service delivery when external actors withdraw.^5^ Refugee-led organizations remain underfunded yet are uniquely positioned to deliver contextually relevant care and to build sustainable pathways forward.^5^ Policymakers and donors should prioritize direct funding to these organizations as both an immediate support and a long-term strategy for self-reliance.

Furthermore, policy reform must extend beyond health to include economic strategies that integrate refugees into civilian labor markets, facilitate access to urban and regional markets, and expand opportunities for income generation.^37^ Uganda’s progressive policies, which allow refugees access to land and services, remain commendable but insufficient without economic integration.^37^ Nakivale’s geographic isolation and concentrated poverty mean that refugees’ skills, whether agricultural, technical, or professional, cannot generate income in markets with little demand.^37^ This structural reality locks refugees into dependence on external aid and renders them unable to pay for services, medications, or transport when funding is cut. Skills training and financial literacy training must be matched to real market opportunities, and integration into surrounding economies should be prioritized so that refugees can begin to bridge the gaps left by fluctuating aid streams.^38^

### Limitations

This study has several limitations. Although compensation was low in comparison to participants’ professional salaries, it is possible that the participation reward might have attracted practitioners with greater financial need or those most motivated to voice grievances, potentially skewing the represented views. Social desirability bias is possible, although the consistency and specificity of accounts across cadres and facilities mitigate this concern. Additionally, the rapidly evolving policy environment means that services may have been restored or further reduced after data collection. Anecdotal reports from health care practitioners indicate that, since data collection, the government has assumed responsibility for hiring and sustaining health care practitioners previously supported by the International Medical Team but has not necessarily retained the same individuals or maintained the same staffing levels, causing another wave of transitions. However, we believe this study has substantial value by capturing the perspectives of Ugandan practitioners actively staffing Nakivale facilities, presenting how macro-budget decisions degrade service delivery and patient outcomes.

## Conclusions

In this qualitative study, health care practitioners reported that the withdrawal of humanitarian aid in Uganda has precipitated a cascading systems crisis: essential services curtailed, supplies and fuel constrained, staff depleted and distressed, and households pushed past subsistence. Nakivale practitioners’ testimonies mirror national and global projections but add vital detail on daily realities and intervention opportunities. Restoring financial flows, securing medical supplies, stabilizing staffing, and leveraging community-based incentives could mitigate the most severe impacts. Documenting both harm and resilience is essential for accountability and for directing scarce resources toward saving lives. Aid decisions should therefore be seen not as distant acts of charity, but as investments in shared stability, security, and human dignity—recognizing that refugee health is central, not peripheral, to global responsibility.

## Notes

Supplement 1. eAppendix 1. Healthcare Provider Interview Guide eAppendix 2. Dropped Codes Due to Low Numbers

Supplement 2. Data Sharing Statement
