# Supplementary material for: Global Aid Cuts and Local Health Consequences in Nakivale Refugee Settlement, Uganda
Source: JAMA Netw Open. 2026 May 26;9(5):e2615000. doi: 10.1001/jamanetworkopen.2026.15000 (PMC13213520; doi:10.1001/jamanetworkopen.2026.15000)
Supplement: Supplement 1. — eAppendix 1. Healthcare Provider Interview Guide eAppendix 2. Dropped Codes Due to Low Numbers [file jamanetwopen-e2615000-s001.pdf]

Supplemental Online Content

Lee H, Bjelland C, Wallace K, Asiimire D, Mwesigwa FS. Global aid cuts and local health consequences in Nakivale Refugee Settlement, Uganda. *JAMA Netw Open*. 2026;9(5):e2615000. doi:10.1001/jamanetworkopen.2026.15000

eAppendix 1. Healthcare Provider Interview Guide

eAppendix 2. Dropped Codes Due to Low Numbers

This supplemental material has been provided by the authors to give readers additional information about their work.

© 2026 Lee H et al. *JAMA Network Open*.

eAppendix 1. Healthcare Provider Interview Guide

1) **Can you describe your overall experience working as a healthcare provider in Nakivale refugee settlement?**
   Probes: What brought you to work here? What do you find rewarding or challenging? How has your role evolved over time?

2) **Since the beginning of 2025, have you noticed any changes in the settlement or healthcare environment?**
   Probes: Consider changes in resources, funding, infrastructure, staffing, policies, or external partnerships.
   - Specific changes related to available contraceptive methods/resources to deal with side effects (pads, pain meds, lubes, etc)
   - What type of methods are the most common method?
   - Is there a specific protocol to follow when women complain about contraceptive side effects?
   - Changes in per head money/food distribution for families?
   - Changes in food/resources for ANC participants?

3) **If yes, can you describe the types of changes you've observed?**
   Probes: Were the changes related to resource availability, staffing patterns, or healthcare service availability?

4) **If yes, how have these changes affected the care you provide to patients? Anything specific to maternal health?**
   Probes: Have you had to modify your clinical practices? Are there new barriers or facilitators to care? Has workload increased or decreased? Changes in prevalent health conditions?

5) **If yes, have these changes influenced the way your health facility or team operates?**
   Probes: Any changes in teamwork, referral systems, communication, or decision-making processes?

6) **If no, do you foresee any changes in the near future? How might those changes impact patient care?**

   Probes: Related to resource availability, staffing patterns, or healthcare service availability?

7) **What do you anticipate will happen in the coming months or years in Nakivale?**
   Probes: Do you expect more changes in patient volume, disease patterns, funding, or your own role?

8) **What would you need to continue providing quality care in the face of ongoing or future changes?**
   Probes: Think about training, resources, staffing, infrastructure, or external support.

9) **Is there anything else you'd like to share about your experience working in a humanitarian context or how things have changed over time?**

Thank the participant for their time and input.

© 2026 Lee H et al. *JAMA Network Open*.

eAppendix 2. Dropped Codes Due to Low Numbers

- Long wait time: 3
- Non-communicable disease burden: 2
- Service quality decrease: 2
- Building space limited: 4
- Mental health exacerbated: 4
- Education quality decrease: 1
- Receiving health center overburdened: 1
- Community attempting to bridging the gap: 1
- Research capacity decrease: 1
- Other barriers to care: 1

Codes that were dropped because their content did not refer to funding cuts:

- Referral services between health facilities limited
- Overall experience in Nakivale
- Attitude towards refugees generally
- Site descriptions
- Newly arrived refugees' knowledge of Nakivale's services

© 2026 Lee H et al. *JAMA Network Open*.
