# Supplementary material for: Global Aid Cuts and Local Health Consequences in Nakivale Refugee Settlement, Uganda
Source: JAMA Netw Open. 2026 May 26;9(5):e2615000. doi: 10.1001/jamanetworkopen.2026.15000 (PMC13213520; doi:10.1001/jamanetworkopen.2026.15000)
Supplement: Supplement 2. — Data Sharing Statement [file jamanetwopen-e2615000-s002.pdf]

# Data Sharing Statement

Lee. Global Aid Cuts and Local Health Consequences in Nakivale Refugee Settlement, Uganda. *JAMA Netw Open*. Published May 26, 2026.
doi:10.1001/jamanetworkopen.2026.15000

**Data**
**Data available:** No

**Additional Information**
**Explanation for why data not available:** We will share the original de-identified qualitative data upon reasonable request.
